# First report of tinea corporis caused by Trichophyton quinckeanum in Iran and its antifungal susceptibility profile

**DOI:** 10.32598/CMM.2023.1344

**Published:** 2022-12

**Authors:** Seyed Reza Aghili, Raheleh Alehashemi, Mahdi Abastabar, Farhad Niknejad, Iman Haghani, Fatemeh Livani, Amineh Kashiri, Javad Javidnia, Mohsen Nosratabadi

**Affiliations:** 1 Invasive Fungi Research Center, Communicable Diseases Institute/Department of Medical Mycology, School of Medicine, Mazandaran University of Medical Sciences, Sari, Iran; 2 Student Research Committee, School of Medicine, Mazandaran University of Medical Sciences, Sari, Iran; 3 Department of Medical Laboratory Sciences, School of Para Medicine, Golestan University of Medical Sciences, Gorgan, Iran; 4 Department of Internal Medicine, Dermatology Group, School of Medicine, Golestan University of Medical Sciences, Gorgan, Iran; 5 Student Research Committee, School of Medicine, Mazandaran University of Medical Sciences, Sari, Iran

**Keywords:** Antifungal susceptibility profile, *T. quinckeanum*, Tinea corporis

## Abstract

**Background and Purpose:**

*Trichophyton quinckeanum*, a known zoophilic dermatophyte responsible for favus form in rodents and camels, is occasionally reported to cause human infections.

**Case Report:**

This study aimed to report a case of tinea corporis caused by *T. quinckeanum* that experienced annular erythematous pruritic plaque with abundant purulent secretions. In June 2021, a 15-year-old girl with an erythematous cup shape lesion on the right wrist bigger than 3 cm in diameter was examined for tinea corporis. Since March, 2016 her family has kept several camels at home. Direct examination of skin scraping and purulent exudates revealed branching septal hyaline hyphae and arthrospore. Morphological evaluation of the recovered isolate from the culture and sequencing of ITS1-5.8S rDNA-ITS2 region resulted in the identification of *T. quinckeanum*. Antifungal susceptibility testing showed that this isolate had low minimum inhibitory concentration (MIC) values for luliconazole, terbinafine, and tolnaftate, but high MICs to itraconazole, fluconazole, posaconazole, miconazole, isavuconazole, ketoconazole, clotrimazole, and griseofulvin. However, the patient was successfully treated with oral terbinafine and topical ketoconazole.

**Conclusion:**

It can be said that *T. quinckeanum* is often missed or misidentified due to its morphological similarity to *T. mentagrophytes*/*T. interdigitale* or other similar species. This dermatophyte species is first reported as the cause of tinea corporis in Iran. As expected, a few months after our study, *T. quinckeanum* was detected in other areas of Iran, in a few cases.

## Introduction

Based on recently modified molecular taxonomy, *Trichophyton quinckeanum*, *Trichophyton schoenleinii*, and *Trichophyton simii* comprise a distinct clade, earlier known as a *T. mentagrophytes*. Var. quinckeanum [ 1 ]. Differentiating between *T. quinckeanum* and *T. mentagrophytes* using conventional methods, such as culture, is challenging and unreliable [ 2 ]. Today, molecular biology methods, such as polymerase chain reaction (PCR) and sequencing of the internal transcribed region (ITS) are used to clearly identify these dermatophytes [ 3 ]. 

This pathogen has been isolated from humans sporadically in Czech, Slovakia, and Germany (Europe) [ 4 , 5 ] and also from rodent and camel in the Middle East (Asia) [ 6 ]. Although previously a case of *T. quinckeanum* isolation was reported from fox in Iran [ 7 ], before the present study, there have been no reports of the isolation of this dermatophyte species from humans in our country. 

Herein, this study reports the first case of tinea corporis due to this organism in Iran. Although, a few months after this study, *T. quinckeanum* was detected in other areas of Iran, in a few cases (available on ). The strain was characterized in terms of clinical findings, patient history, and mycological conventional methods. Moreover, it was subsequently analyzed by PCR-restriction fragment length polymorphism (RFLP) molecular biology method with MvaI enzyme and sequencing of ITS region.

## Case report

A 15-year-old girl living in rural areas of Gonbad city, Golestan province, Iran, had been raising several camels in her family house since 5 years ago. A few weeks before the examination, an erythematous macule appeared on the top of the right wrist, accompanied by a mild itching sensation. During about 3 weeks, the macule gradually increased in size, and a lesion developed with severe itching, scaling, and purulent secretions. 

**Figure 1:**
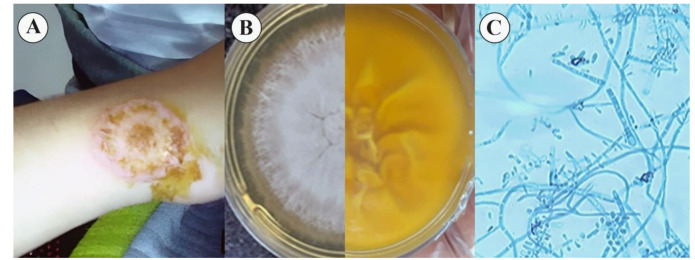
A) Tinea Corporis in the right wrist due to *Trichophyton quinckeanum* B) Color and texture in culture after 15 days on Mycobiotic agar C) Microscopic slide view

She was visited by a dermatologist and was referred to a pathology laboratory. The dermato-logist also recommended a test to diagnose leishm-aniasis in addition to a fungal examination. The purulent exudates obtained from the lesion were examined by Giemsa staining, an assay used to detect Leishman bodies. However, no evidence was found in favor of leishmaniasis. Physical appearance at the time of sampling for mycological assay showed a single annular erythematous pruritic cystic plaque with fluid material which was scaly with active inflamed raised borders and a clear center that grew centrifugally above the right wrist with a diameter of more than 3 cm (Figure. 1A).

The patient was initially treated with hydrocortisone 1% topical ointment (Iran najo pharmaceutical Co., Tehran, Iran) to relieve the itching and povidone Iodine 10% (Aburaihan pharmaceutical Co., Tehran, Iran) by herself. However, the lesion did not disappear and was gradually expanded. Direct examination of scrapings and exudates by 10% potassium hydroxide revealed hyphae and the formation of arthrospores which indicated dermatophytosis. Culture of lesion samples at 30 °C on Mycobiotic agar (Laboratorios Conda S.A. Madrid. Spain) yielded primarily white powdery colony within 5 days, that subsequently became cottony in the center and granular in margin and formed a yellowish-brown reverse pigment colony (Figure 1B) in 15 days. 

Lactophenol cotton blue staining of slide cultures revealed septate hyphae and pencil-shaped, thin-walled macroconidia on conidiophores and numerous pear-shaped microconidia irregularly located around the hyphae (Figure 1C). 

Genomic DNA was extracted from the culture grown for at least 10 days on sabouraud’s dextrose agar (Laboratorios Conda S.A. Madrid. Spain) according to a protocol described by Makimura [ 8 ]. Briefly, PCR was carried out to amplify the ITS Region with ITS1 (5′-TCCGTAGGTGAACCTGCGG-3′) and ITS4 (5′-TCCTCCGCTTATTGATATGC-3′) primers (SinaColon, Iran). 

**Figure 2:**
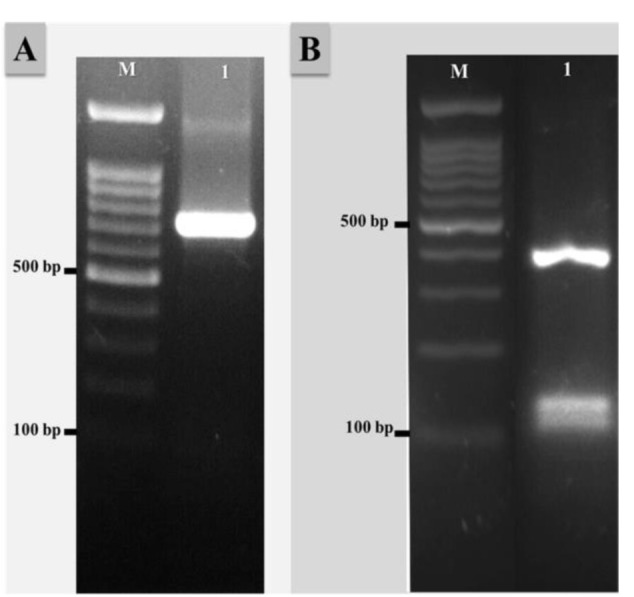
Agarose gel electrophoresis, A) Internal transcribed spacer-polymerase chain reaction products of *Trichophyton quinckeanum* (about 683 bp), B) Polymerase chain reaction-restriction fragment length polymorphism pattern of *T. quinckeanum* (403,124, 103, and 50 bp). M: 100 bp molecular size marker

Amplification was performed based on the following conditions: denaturation at 94 °C for 7 min, followed by 30 cycles at 94 °C for 1 min, 52 °C for 30 s, 72 °C for 60 s, and finally an extension at 72 °C for 7 min. After verification by electrophoresis on 1.0% agarose gels, the PCR products were amplified by about 683 bp (Figure 2A). Initially, PCR production of ITS1-5.8s-ITS2 region of ribosomal DNA (rDNA) was digested by the *MvaI* restriction enzyme for the identification of the PCR-RFLP pattern. This pattern was very similar to the *T. schoenleinii* (Figure 2B). 

**Figure 3:**
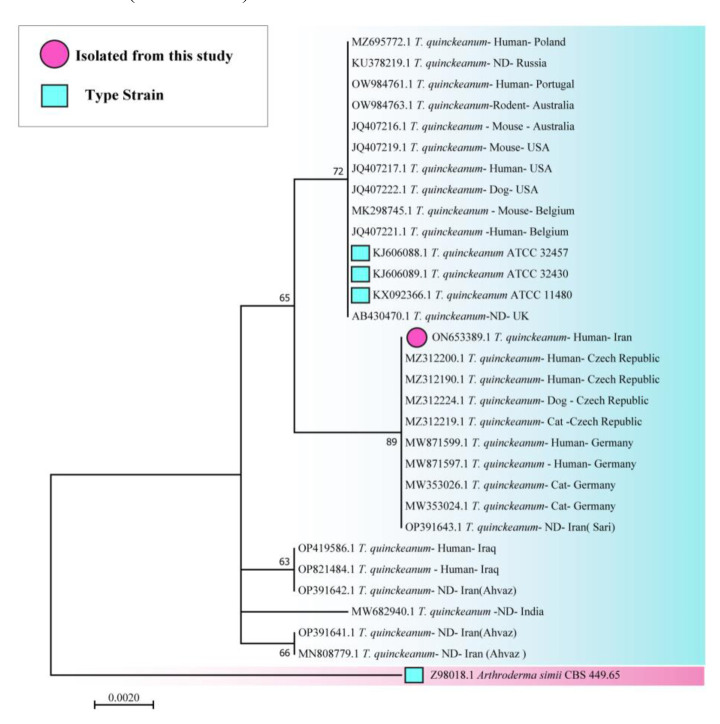
Phylogenetic tree of 30 representative *Trichophyton quinckeanum* based on the analysis of the internal transcribed spacer region sequences constructed using the neighbor-joining method.

The ITS amplified product was subjected to sequencing and the obtained sequence was edited with MEGA software (version 11). The strain was identified at the species level by BLASTN searches of the sequence against the National Center for Biotechnology Information (NCBI) Nucleotide (). The sequence was submitted to GenBank and registered with the accession number ON653389 and compared with the reference sequences deposited in GenBank in NCBI databases. The results showed 100% similarity to the *T. quinckeanum* strain with the accession number MZ31200.1. Finally, a phylogenetic tree was constructed with the neighbor-joining method using MEGA software (version 10.1) and 1000 bootstraps. *Arthroderma simii* (Z98018.1) was used as an out-group (Figure 3).

The genomes of our isolated strain (ON653389.1) are similar to the other strain (OP391643.1) isolated from Mazandaran province after this study. For this species, using 14 antifungal agents, in vitro susceptibility test was performed according to the broth microdilution assay described in the Clinical and Laboratory Standards Institute M38-A3 document [ 9 , 10 ].

Afterward, based on direct examination, clinical findings, molecular identification, and in vitro susceptibility test, the treatment was switched to oral terbinafine 250 mg/day (Pars Darou pharmaceutical co. Tehran, Iran) and ketoconazole topical cream 2% (Kish-Medipharm Co., Kish,Iran) applied twice daily for three weeks. In addition, betamethasone-Najo 1% topical ointment (Iran najo pharmaceutical Co., Tehran, Iran) was used to reduce inflammation in the lesion. The lesion disappeared within a month and has not relapsed.

The case in this study was diagnosed with tinea corporis caused by *T. quinckeanum*. The findings indicated that *T. quinckeanum* was highly susceptible to luliconazole (minimum inhibitory concentration [MIC] range: <0.001 µg/ml), terbinafine (MIC range: <0.016 µg/ml), and tolnaftate (MIC range: <0.032 µg/ml). It should be mentioned that the highest MIC value was observed for fluconazole (32 μg/ml). Moreover, the MIC was obtained for butenafine (0.125 μg/ml), voriconazole (0.125 μg/ml), amphotericin B (0.125 μg/ml), econazole (0.5 μg/ml), griseofulvin (2 μg/ml), clotrimazole (4 μg/ml), isavuconazole (4 μg/ml), ketoconazole (4 μg/ml), miconazole (8 μg/ml), and itraconazole (8 μg/ml).

## Discussion

Dermatophytes usually spread effectively only in their main host(s), and transmission to other hosts rarely occurs [ 11 ]. It must be noted that *T. quinckeanum* is historically connected to rodent favus [ 12 ] and camel dermatophytosis [ 6 ] typically. The infection in domestic animals occurs due to transmission from rodents which is rarely transmitted to humans. Human infection due to this species is associated with low social and economic status and poor hygiene [ 13 ]. 

In recent decades, the health situation in developing countries has improved. Therefore, this species has been only sporadically isolated from humans in Europe, Africa, and the Middle East. This fungus had not been isolated from humans in Iran and this is the first case of tinea corporis due to *T. quinckeanum* in the north of Iran and southeast of the Caspian Sea, which is most likely contracted from a camel. Since this organism was previously isolated from a fox in other regions of Iran, we believe that there is a possibility of contracting the disease from it in other regions of our country. 

This species is often missed or misidentified due to its morphological similarity to *T. mentagrophytes*/*T. interdigitale* or other superficially similar species [ 14 , 15 ]. Jackson et al. separated 17 dermatophyte species from each other by PCR-RFLP method of ITS-rDNA regions with MvaI restriction endonuclease. However, the MvaI restriction enzyme cannot distinguish *T. quinckeanum* and *T. schoenleinii* from each other, while the taxon pairs are closely related [ 16 ]. 

An important step for the identification of fungal pathogens is to align them into groups of organisms with common molecular methods, which has become the main basis of phylogenetic studies. A phylogenetic tree is a branching diagram showing the evolutionary relationships among various species based on similarities and differences in their physical or genetic characteristics [ 17 ]. 

In this study, the identification of isolated dermatophyte was confirmed using sequencing of the ITS region of rDNA. The dendrogram of this species showed genetic differentiation between *T. quinck-eanum* and the closely related species, *A. simii*. Moreover, this dendrogram revealed the distinction of all newly described genotypes within the species. 

Two strains of *T. quinckeanum* isolated from the north of Iran (our strain and OP391643.1 that isolated in Sari, Mazandaran, a neighboring province) were closely similar. However, they differed from the three isolated stains in Khuzestan (a southwest province of Iran). It showed that environmental exposures can change gene expression through genetic and epigenetic mechanisms [ 18 ]. 

Only a few studies have been performed on *T. quinckeanum* in the field of antifungal susceptibility testing. However, it has been found that luliconazole had the lowest MIC value for *T. quinckeanum*, but this drug has been approved as a 1% topical cream in Japan and USA [ 19 ] and is not widely available in Iran yet. In line with the results of the present study, Łagowski et al. [ 20 ] found that *T. quinckeanum* was highly susceptible to allylamines, particularly terbinafine in broth microdilution test, and most patients were treated with this antifungal medication. 

Terbinafine and tolnaftate demonstrated low MIC values in comparison with the other tested antifungals. *T. quinckeanum* infection was usually treated with different systemic and topical antifungal drugs and mainly a combination of antifungal drugs [ 11 ]. In the present study, the patient also recovered by using oral terbinafine along with ketoconazole cream; however, this organism showed high MICs to ketoconazole. Determination of the most appropriate medications for the treatment of diseases caused by an organism requires large-scale clinical trials.

It must be mentioned that we have no clear explanation of how *T. quinckeanum* infection was transmitted that could be substantiated by data. However, since some studies [ 6 , 21 ] have mentioned camels as the main natural reservoirs of this organism, a possible hypothesis is that the infection of the patient was due to contact with camels. The spread among humans may be caused by a shift in the virulence and other biological properties of the pathogen. 

In this study, the infection was found in a 15-year-old female. This can probably be explained by the fact that girls in rural areas are more active in the livestock breeding process. Uhrlaß et al. [ 6 ] also found this dermatophyte species more in females, compared to males. They also stated that 43% of the patients were children and adolescents (≤19 years of age) and cats were a frequent source of infection.

## Conclusion

This study presented the first case of human tinea corporis due to *T. quinckeanum* in Iran. It was discovered that this zoophilic dermatophytes species can be found in different hosts. Its identification from similar species based on the type of lesions, the morphology of the fungus in the culture medium, and even PCR-RFLP with Mva1 enzyme, which is used for the identification of many dermatophytes, is not possible. Therefore, other molecular methods must be employed, including sequencing of the ITS-rDNA region. Results of this study also revealed that the combination therapy with systemic terbinafine and topical antifungal creams will remove the infection due to this dermatophyte species.

## Acknowledgments

The authors would like to thank the patient, her family members, and the staff of the *Leishmania* Research Laboratory, Gonbad Kavus Health Center, especially Ms. Fatemeh Mesgarian for cooperation with this study.

## Authors’ contribution

SR.A. designed, wrote, and reviewed the paper. R.A. performed sampling, data collection, and myco-logical identification. M.A. edited and revised the study. F.N., I.H., and J.J. cooperated in molecular identification. F.L. and A.K. performed the clinical and therapeutic study. M.N. cooperated in the antifungal susceptibility testing. All authors read and approved the final manuscript.

## Conflicts of interest

None of the authors have any conflicts of interest to declare.

## Financial disclosure

This research received no specific grant from any funding agency in the public, commercial, or not-for-profit sectors.

## Ethical considerations

This case report was performed in compliance with the Declaration of Helsinki and approved by the Ethics Committee of Mazandaran University of Medical Sciences, Sari, Iran (IR.MAZUMS.REC.1399.8455)
